# Synthesis of Hydroxyapatite-Gelatin Composite Hydrogel for Bone Tissue Application

**DOI:** 10.3390/gels11080630

**Published:** 2025-08-10

**Authors:** José Luis Barrera Bernal, Íñigo Gaytán Salvatella, Bryan Iván Martín del Campo, Marco Antonio Alvarez Perez, David Masuoka-Ito

**Affiliations:** 1 Biomaterials Research Laboratory, Department of Stomatology, Health Sciences Center, Autonomous University of Aguascalientes, Aguascalientes City 20100, Mexico; joseluis.barrera@edu.uaa.mx; 2 Tissue Bioengineering Laboratory, Postgraduate Studies and Research Division, Faculty of Dentistry, National Autonomous University of México (UNAM), México City 04510, Mexico; igaitansalvatella@gmail.com (Í.G.S.); marcoalv@unam.mx (M.A.A.P.); 3 Department of Prosthodontics, Graduate School of Dentistry, Showa Medical University, Tokyo City 145-8515, Japan; bryan.mctellez@gmail.com

**Keywords:** hydrogel, hydroxyapatite, composite material, biocompatibility, bone tissue

## Abstract

Bone tissue engineering has gained attention recently as a method for regenerating bone critical-size defects. This work aims to synthesize a hydrogel based on gelatin, di-amine polyethylene glycol, Polyethylene Glycol-Polypropylene Glycol-Polyethylene glycol, using genipin as a cross-linker and adding hydroxyapatite as a ceramic insert that can be used as a cellular scaffold in bone tissue engineering. Characterization was performed using Fourier transform infrared spectroscopy, identifying the leading absorption bands to verify that the hydrogels cross-linked correctly. The hydrogels with elastic modules and resistances that best adapted to the values reported for the mandibular trabecular bone were identified through mechanical tests. Using scanning electron microscopy, the presence of hydroxyapatite in the hydrogels was verified. The hydrogels with the best results were selected to carry out the biological assays. The cell viability assay verified that the osteoblastic cells proliferated better in the hydroxyapatite scaffolds, and the composite hydrogel induced osteoblast differentiation from undifferentiated mesenchymal stem cells. Hydrogels loaded with hydroxyapatite proved to be a promising biomaterial with potential application in bone regeneration.

## 1. Introduction

Bone is a hierarchical connective tissue integrated as a composite material. It comprises organic components like collagen fibrils that provide tensile strength, with inorganic components like nano-hydroxyapatite crystals, responsible for hardness and rigidity [1,2]. Bone can be affected by several pathologic processes like tumors, cysts, and trauma; however, it can regenerate itself, except for particular defects that, due to their size, cannot repair themselves, called critical-size bone defects. These defects disrupt the body’s natural ability to repair bones, and therefore, the regeneration of critical-sized bone defects remains a pressing challenge in orthopedics and the dental regeneration field [3]. Several strategies have been proposed for these defects: Traditional approaches like autografts, where bone tissue is harvested from the patient, offer excellent osteoconductive properties (bone cell growth promotion) but come with limitations, including donor site morbidity, pain, and limited availability of suitable donor tissue [4]. Allografts utilize bone tissue from a different human donor and can address some limitations, such as disease transmission, immune rejection, and extensive tissue processing. Xenografts, harvested from animal sources like cows or pigs, offer theoretical advantages regarding availability but have significant drawbacks; they can trigger immune responses and raise some ethical concerns [4,5].

Due to these limitations, researchers are actively exploring alternative strategies with biomaterials for bone regeneration. Tissue engineering is a multidisciplinary field combining engineering and life sciences principles to develop biological substitutes that restore, maintain, or improve tissue function. In bone regeneration, tissue engineering offers a promising approach to addressing critical-size defects and enhancing natural healing. One of the significant advancements in tissue engineering is using 3D biomaterials because they better mimic the in vivo environment, allowing for improved cell–cell and cell–matrix interactions. This setup more accurately resembles the natural cellular microenvironment, enhancing physiological relevance and improving the predictability of in vivo responses [6].

One promising area in bone tissue regeneration is the use of hydrogels. Hydrogels are considered 3D natural or synthetic polymeric scaffolds that are highly hydrophilic and biocompatible, able to absorb up to several times their dry weight in water, causing swelling of their morphology without causing disintegration, making them ideal for bone tissue applications [7,8]. Hydrogel materials have recently been explored for bone tissue regeneration [9,10]. Hydrogels can be classified as natural or synthetic [11]. Natural hydrogels can be based on polysaccharides like alginate, chitosan, and hyaluronic acid or on proteins such as collagen and gelatin [11,12]. Those materials are well-degraded and biocompatible but have lower mechanical properties. Hydrogel materials can be photopolymerized, allowing for the control of properties such as porosity, graft architecture, and mechanical properties, which can be tailored to different functions and applications. Di-amine polyethylene glycol (PEG-D) is a synthetic material used to synthesize hydrogels; the hydroxyl functional groups of the PEG molecule are replaced by diamine groups, which can be easily cross-linked by attack of the ring in the genipin molecule and finally produce genipin oligomers that bind to the polymer’s network [13]. Polyethylene Glycol, Polypropylene Glycol, and Polyethylene Glycol (PEG-PPG-PEG) are poloxamers known as Synperonic or Pluronic. Poloxamers are a group of symmetrical triblock copolymers that are characterized by their amphipathic nature, as they have two hydrophilic polyethylene glycol (PEG) molecules at the ends, while in the center is the polypropylene glycol (PPG) that contains a methyl group of hydrophobic nature [13].

The synthesis of composite hydrogels has gained attention in bone tissue regeneration due to their ability to mimic the natural environment of the extracellular matrix and provide mechanical support for cell adhesion, migration, and proliferation [14]. However, excessive water gain must be controlled because hydrogels with excessive water gain can exhibit minimal or no intrinsic biological activity due to the non-adhesive nature of the PEG chains [15]. This occurs because the hydrogel forms a hydrated surface that inhibits the adsorption of specific adhesion proteins such as integrins and cadherins [14,15].

The mechanical properties of hydrogels make them inadequate for bone tissue, even in non-load-bearing zones [16]. In addition, the excessive water gain makes the hydrogel even weaker. However, specific to bone tissue, elastic modulus and tensile strength can be custom-made by controlling the cross-linking process [17]. Therefore, their properties can also be tailored to regulate cross-linking and meet different needs [7,14,15,16,17,18,19]. Control cross-linking means controlling degradation rates, mechanical properties, swelling, and controlled release, thus meeting specific needs [14]. The gold standard cross-linking method is glutaraldehyde (GA), a saturated dialdehyde used as a high-level disinfectant. Unfortunately, this cross-linker has been reported as highly cytotoxic [20,21]. Genipin, which appears as an alternative to GA, is an aglycone, a biodegradable molecule obtained from the Gardenia jasminoides with low cytotoxicity that has been proposed as a natural cross-linker [22]. Genipin reacts with amine groups (–NH_2_) presented in the gelatin backbone to form covalent bonds via Schiff base in a slow reaction (two-step reaction) characterized by nucleophilic attack [22].

Furthermore, the softness of the hydrogel for bone tissue allows for searching ceramic filling materials, such as hydroxyapatite, that can be added to the hydrogel, improving stiffness and mechanical performance for load-bearing applications. Hydroxyapatite, a ceramic filler for bone scaffolds, has been utilized in engineering applications due to its osteogenic, osteoconductive, and osteoinductive properties, improving hydrogel biomechanical properties and cell behavior [23]. The synthesis of composite hydrogels has gained attention in bone regeneration due to their ability to mimic the natural environment of the extracellular matrix and provide mechanical support for cell adhesion, migration, and proliferation; moreover, various composite hydrogel materials have been explored for bone tissue regeneration in recent years, including alginate, chitosan, hyaluronic acid, and gelatin, which exhibit enhanced mechanical performance, allow cell infiltration, stimulate mesenchymal stem cells and osteoblast differentiation, and bone formation [24,25,26,27]. The relevance of this work is the development of a composite hydrogel that functions as a cell scaffold based on gelatin, di-amine polyethylene glycol (PEG-D), Polyethylene Glycol-Polypropylene Glycol-Polyethylene glycol (PEG-PPG-PEG), using genipin as a non-toxic cross-linker, and adding hydroxyapatite (HAp) as a filling inorganic ceramic material that enhances the mechanical properties, providing a simple method for synthesizing a hybrid hydrogel scaffold that promotes cell biocompatibility and cell bioactivity for tissue engineering applications.

## 2. Results and Discussion

### 2.1. Gelatin/HAp Hydrogels Synthesized

Hydrogels were prepared with an overall polymeric content of 17% (*w*/*v*) based on a previous study [28]. Some of the hydrogels were crosslinked with glutaraldehyde, and some with genipin at different concentrations. Different concentrations of HAp were also tested. These variations were performed to examine the effect of the physical, chemical, mechanical, and biological changes in the hydrogels.

### 2.2. Mechanical Behaviour of Hydrogels

**Figure 1:**
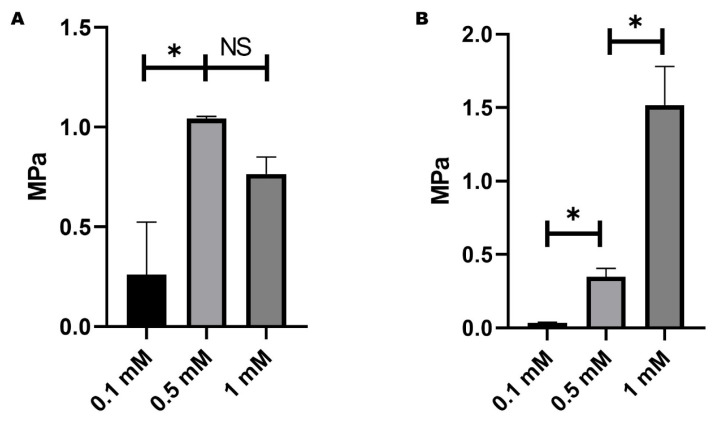
Mechanical test for optimal concentration of genipin hydrogel scaffold. (**A**) Elastic modulus of the gelatin hydrogels with three concentrations of genipin. (**B**) Tensile strength of the gelatin hydrogels with three concentrations of genipin. Asterisks (*) indicate significant differences at *p*-value < 0.05. NS means no significant differences; error bars show standard deviation.

The mechanical test determined the ideal genipin concentration according to the standard curve (Figure 1). Three concentrations were tested (0.1, 0.5, and 1 mM). In Figure 1A, an elastic modulus is shown, and it can be inferred that more stiffness is acquired at higher concentrations. Simultaneously, increasing the cross-linker amount in the samples improves the tensile strength; however, there were no statistically significant differences in elastic modulus (Figure 1A) between 0.5 and 1 mM; hence, 0.5 mM concentration was selected as the ideal concentration for the rest of the test because the 1 mM concentration induces an intense blue color on the samples.

**Figure 2:**
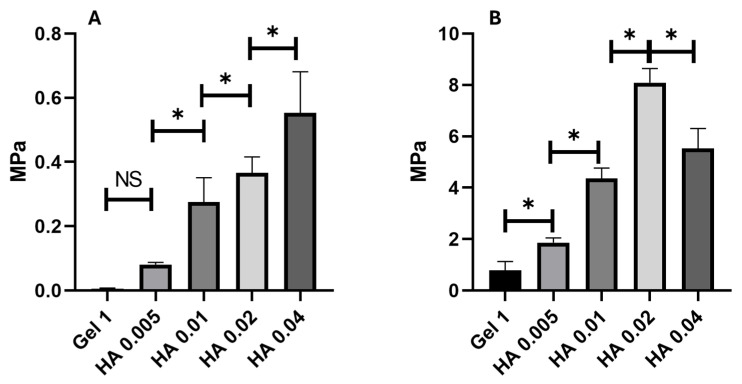
Mechanical test for optimal concentration of Hydroxyapatite as the dopant in the hydrogel scaffold. (**A**) Elastic modulus of the gelatin hydrogels with different concentrations of HA for a standard curve. (**B**) Tensile strength of the gelatin hydrogels with different concentrations of HA for a standard curve. Asterisks (*) indicate significant differences at *p*-value < 0.05. NS means no significant differences; error bars show standard deviation.

As a dopant hydrogel, four concentrations of bioceramic were mechanically tested to choose the optimal concentration of HAp (0.005, 0.01, 0.02, and 0.04 g). The different concentrations of HAp make the hydrogel stiffer and tougher, respectively; however, statistical differences were found between 0.02 and 0.04 g concentrations, making the 0.04 g concentration weaker, as seen in Figure 2. Therefore, we selected 0.02 g of HAp as the final concentration for the rest of the mechanical test, because a higher concentration induces a decrease in tensile strength.

**Figure 3:**
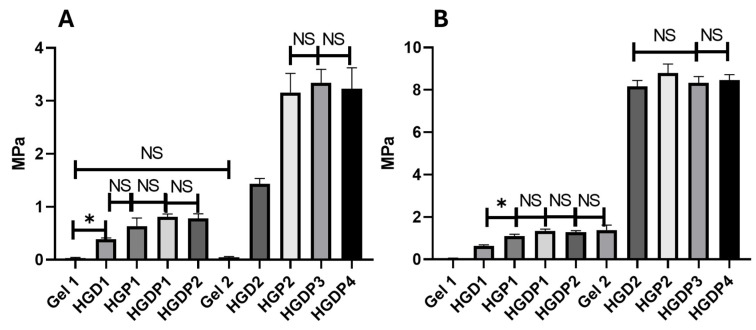
Mechanical assay of hydrogel with 0.5 mM of genipin and 0.02 g of HAp. (**A**) Elastic Modulus of the hydrogels. (**B**) Tensile Strength of the hydrogels. Asterisks (*) indicate significant differences at *p*-value < 0.05. NS means no significant differences; error bars show standard deviation.

In Figure 3, all the hydrogels had the same concentrations of genipin (0.5 mM) and HAp (0.02 g), except for Gels 1 and 2, which were only cross-linked gelatin that served as a control. As can be appreciated, genipin makes the hydrogels stiffer; statistical differences were found between HGD1 and HGD2, HGP1 and HGP2. The stiffer hydrogels were HGP2, HGDP3, and HGDP4 (Figure 3A). Moreover, hydrogels HGD2, HGP2, HGDP3, and HGDP4 had the highest tensile strength values, as shown in Figure 3B.

### 2.3. Hydrogel Structure and Morphology

**Figure 4:**
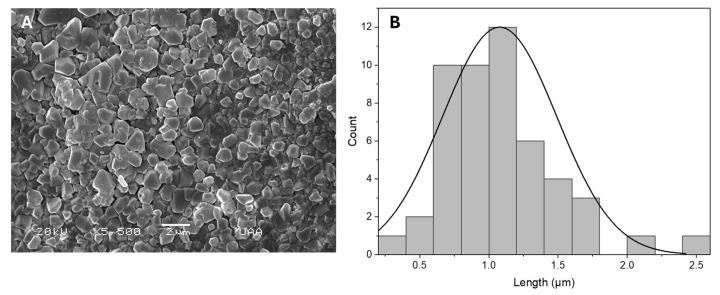
Hydroxyapatite structural morphology (**A**) and histogram of particle size (**B**).

The particles’ hydroxyapatite (HAp) morphology had an irregular prism form and measured 1.07 μm in diameter, as shown in Figure 4.

**Figure 5:**
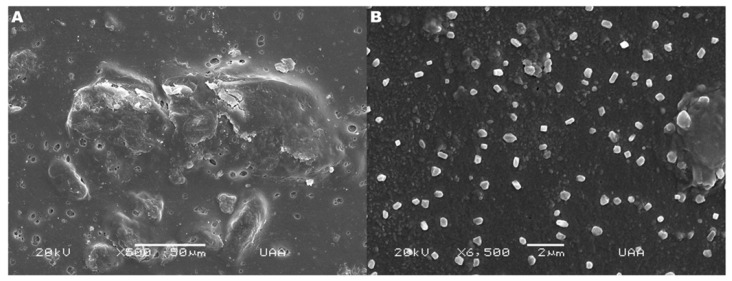
SEM images of the composite hydrogel. (**A**) Micropores inside macropores can be observed. (**B**) SEM micrograph of the synthesized hydrogel with the HAp. Particles along the hydrogel surface can be appreciated.

The morphology and structure of the HAp hydrogel were analyzed by SEM (Figure 5). The hydrogel showed a porosity where macropores and micropores could be observed within the hydrogel structure (Figure 5A). Meanwhile, the hydroxyapatite particles could be appreciated along the surface of the hydrogel, closely adhering to all the morphology, allowing us to observe that the particles were not modified during the synthesis process of the composite hydrogel (Figure 5B).

### 2.4. Characterization

#### 2.4.1. FTIR Analysis Findings

FTIR analysis of the hydrogels, gelatin powder, and cross-linkers was conducted separately to identify new bands on the hydrogel graphs, indicating the formation of bonds between gelatin and glutaraldehyde or genipin.

**Figure 6:**
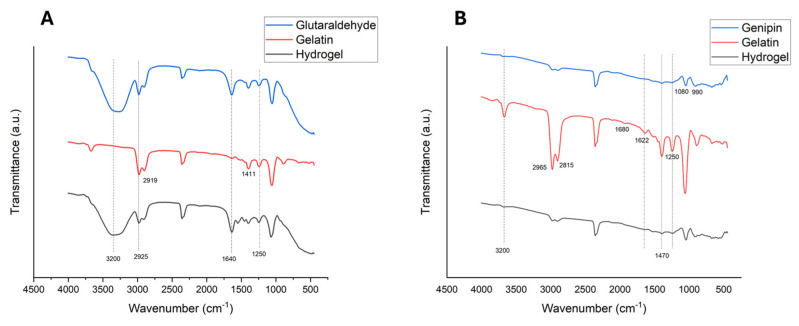
(**A**) FTIR showing the principal absorption bands of the gelatin hydrogel cross-linked with glutaraldehyde. (**B**) FTIR shows the principal absorption bands of the gelatin hydrogel cross-linked with genipin.

The glutaraldehyde FTIR Figure 6A found a typical 3200 cm^−1^ band corresponding to the stretch O-H vibration. Carbonyl C=O stretching on 1716 cm^−1^ and C-H stretching on 2925 cm^−1^ corresponding to the glutaraldehyde molecule were registered. Also, the 2925 cm^−1^ band of the glutaraldehyde molecule persists on the cross-linked hydrogel [29]. An asymmetric stretching vibration in 2919 cm^−1^ for -CH_2_- can be appreciated in the unreacted gelatin spectrum, also δ(C-O) vibration of symmetrical carboxylate groups (COO^−^) can be found in 1411 cm^−1^. The principal vibration bands that correspond to Amide I (C=O stretching), Amide II (NH bending), and Amide III on 1600–1800 cm^–1^, 1470–1570 cm^–1^, 1250–1350 cm^–1^ can be found in Figure 6A, respectively.

In the genipin molecule analysis (Figure 6B), can be observed the O-H stretching that corresponds to water in 3200 cm^−1^, 1080 cm^−1^ (C-H ring in-plane bend), and 990 cm^−1^ (C-H ring out-of-plane bend) characteristics of unreacted genipin were found [30]. Stretching vibrations for the carboxymethyl group (C=O) and aromatic ring (C=C) were found at 1680 and 1622 cm^−1^, respectively [31]. On the genipin cross-linked hydrogel analysis, the persistence of the Amide I (1680 cm^−1^) and Amide II bands (C-N vibration at 1470 cm^−1^) can be noticed. However, in Amide III, a shift can be appreciated in 1250 cm^−1^ that corresponds to a Schiff base, which confirms the cross-linking capacity of genipin.

#### 2.4.2. Equilibrium Swelling Ratio Results

**Figure 7:**
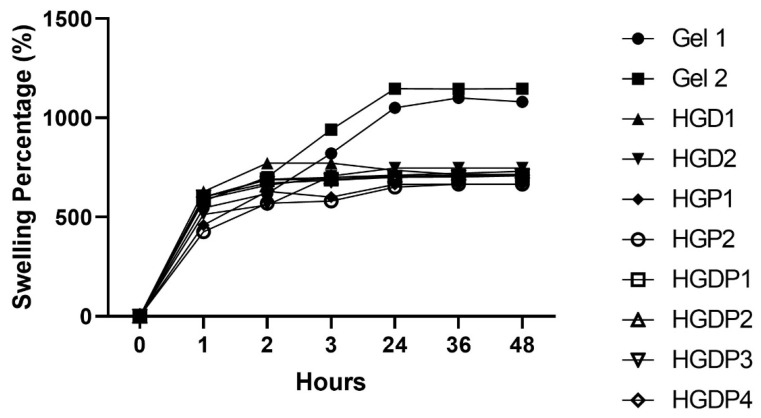
Equilibrium swelling ratio of the hydrogel samples.

In evaluating the equilibrium swelling ratio, all hydrogels obtained a significant gain in water (Figure 7). However, the groups that reported greater gain of water were Gel 1 and Gel 2; hence, gelatin cross-linked with glutaraldehyde and genipin, and the rest of the hydrogels had a similar behavior. All the samples stop gaining water between 24 and 35 h until they reach a plateau in the graphs.

#### 2.4.3. Swelling Percentage

**Figure 8:**
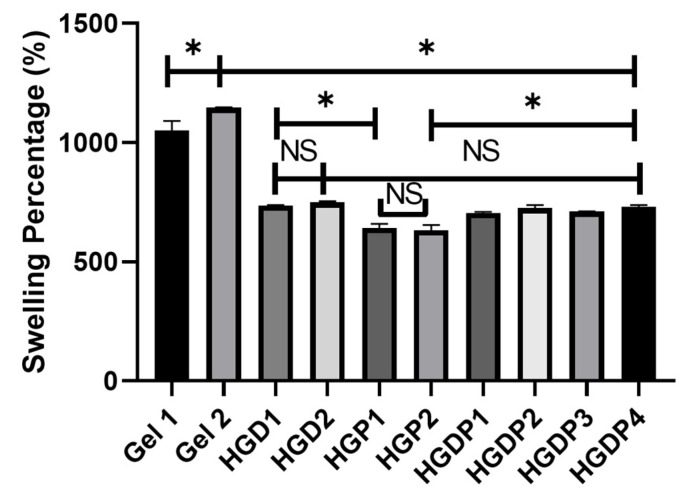
Swelling percentage of the hydrogels. Asterisks (*) indicate significant differences at *p*-value < 0.05. NS means no significant differences; error bars show standard deviation.

Once the equilibrium swelling ratio was calculated, the swelling percentage was obtained simultaneously for all the groups in Figure 8. Gel 2 had the most significant water gain of all the hydrogels, with statistically significant differences against Gel 1. The hydrogels that had the lowest swelling percentage were the groups that included the Pluronic polymer, which means HGP1 and HGP2.

### 2.5. Biological Characterization Results

#### 2.5.1. Cell Viability Tests

**Figure 9:**
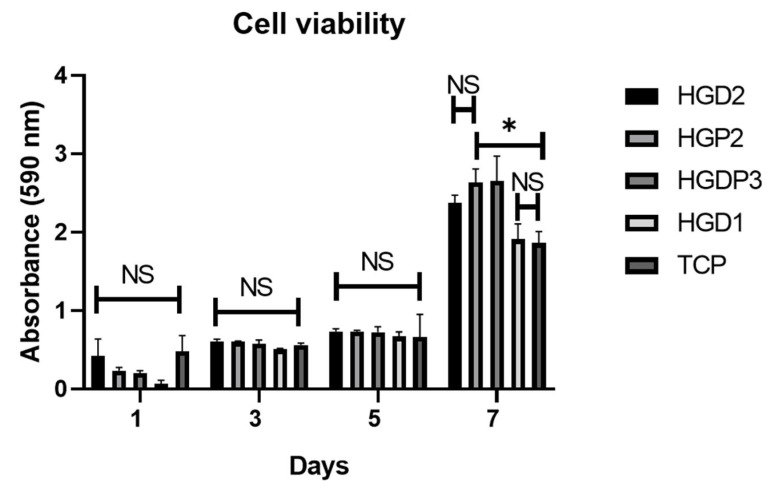
Cell viability of hFOB culture onto the hydrogels at 1, 3, 5, and 7 days. Asterisks (*) indicate significant differences at *p*-value < 0.05. NS means no significant differences; error bars show standard deviation.

A cell viability assay was performed to evaluate the hydrogels’ biocompatibility using the MTT^®^ reagent. As shown in Figure 9, the viability in the day 7 group was significantly higher than on the other days, demonstrating an exponential increase in viability over the course of 7 days. Regarding intergroup comparisons, the HGDP3 and HGP2 groups exhibited higher absorbance values than the other groups, indicating greater cell growth in these groups, which supports the biocompatibility of the hydrogels and confirms that they provide a favorable environment for cell growth.

#### 2.5.2. QRT-PCR Test

**Figure 10:**
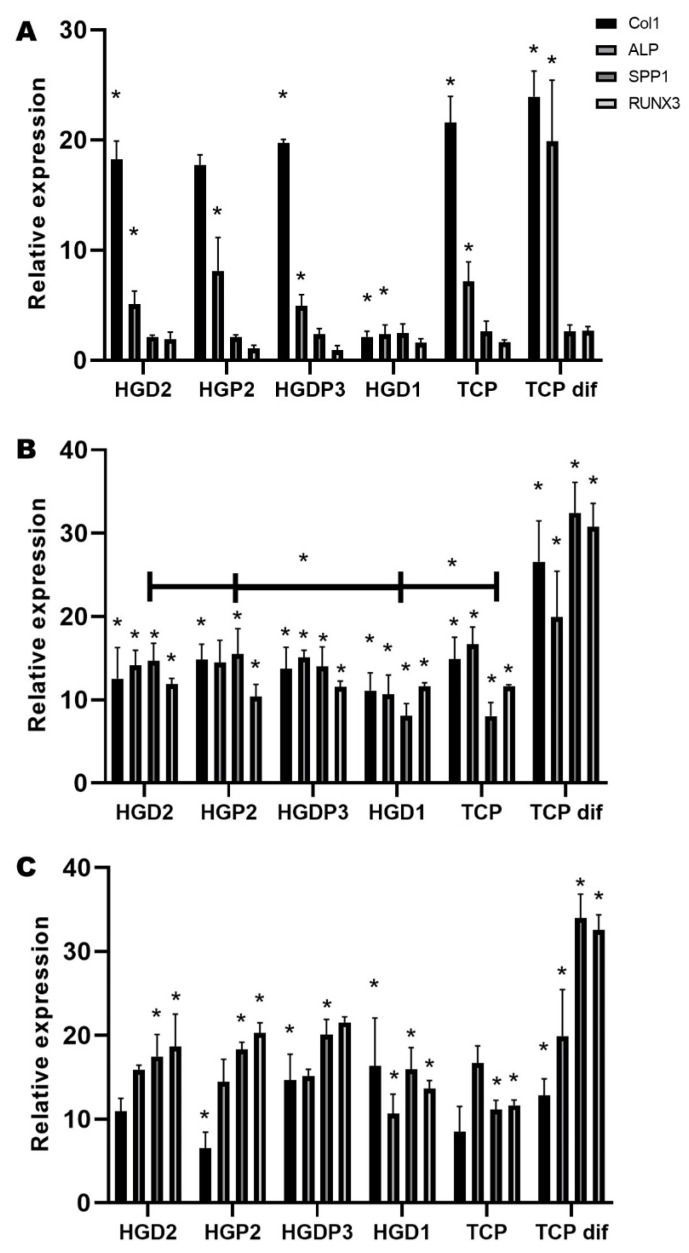
Real-time quantitative polymerase chain reaction (RT-PCR). The histogram showed Col1, ALP, SPP1, and RUNX3 relative expression compared to control TCP and TCP dif (osteogenic media). Results of the expression of representative target genes were normalized against GAPDH. Data represents the mean values of performed triplicates per test sample. (**A**) On day 1, the cells show a relative expression of Col1 and ALP with statistically significant differences. (**B**) At day 7, the activity of SPP1 significantly increased. TCP dif showed the highest expression values. (**C**) On day 30, all the groups showed higher relative expression of markers against the negative control (TCP). The cells with osteogenic media present statistically significant differences in transcripts against the rest of the groups of the evaluated genes in TCP dif. Asterisks (*) indicate significant differences at *p*-value < 0.05.

QRT-PCR was performed to assess the osteogenic differentiation of MSCs up to day 30 and to evaluate the differentiation capacity of these cells cultured onto the hydrogels (Figure 10). As shown in Figure 10A, COL1 (being an early expression gene during osteogenesis) is found to have a higher relative expression compared to the other genes, except for the last group, TCP dif (positive control), where alkaline phosphatase (ALP) begins to be expressed. On day 7 (Figure 10B), it is shown that, as on day 1, the TCP dif group has a higher relative expression compared to the other days, highlighting a significant increase in SPP1 and RUNX3 over COL1 and ALP, as expected for already differentiated MSCs. In the other groups, a higher expression of COL1 and ALP is observed, especially in the groups HGDP3 (hydrogel with gelatin, PEG-D, PEG-PPG-PEG, and genipin), HGD1 (hydrogel with gelatin, PEG-D, and glutaraldehyde), and TCP (plate where MSCs are cultured alone), unlike the others, where there are no significant differences. Finally, on day 30 (Figure 10C), a significant increase in the late expression genes SPP1 and RUNX3 and a decrease in the early expression genes COL1 and ALP are shown, in general in all groups, being more noticeable in the TCP dif group. Moreover, the significant increase in ALP in the TCP group over the other genes stands out. Overall, the qRT-PCR results support the biocompatibility of the hydrogels and their ability to support the osteogenic differentiation of GMSCs. Over time, the differential expression of osteogenic markers suggests that hydrogels provide a dynamic environment that promotes sequential stages of osteogenic differentiation.

The present study demonstrated that the HAp as a ceramic insert could modify the hydrogels’ mechanical properties; as seen in Figure 2, increasing the HAp concentration makes the hydrogel stiffer, as previously reported [17]. Chen et al. also reported that adding 5% of HAp increases Young’s modulus 2 times compared with a scaffold without HA [32]. Ishihara et al. found that genipin made stiffer hydrogels and that stimuli can influence specific cellular responses [33]. Increasing the concentration of HAp makes the hydrogels more resistant (Figure 2B) until a point in the calibration curve where the highest concentration of ceramic insert makes the hydrogel weaker. This is probably because the amount of HAp agglomerates in the polymer network interferes with the cross-linking, producing a more looser polymer matrix, which agrees with the previous report by Lie et al., where an increment of 30 to 40% vegetable-tanned collagen fibers (VCF) in a composite hydrogel of gelatin produces a decrease in mechanical properties [31].

For this improvement in elastic modulus and tensile strength, the electrostatic interaction between HAp and Gelatin is very important. Gelatine is a natural protein rich in amine (-NH_3_^+^), carboxyl (-COO^−^), that can interact with calcium ions (Ca^2+^) and phosphate groups (PO_4_^3−^) presented in the hydroxyapatite [34].

In Figure 3, all the hydrogels had the same concentration of cross-linker and HAp; as can be appreciated, genipin by itself improves the mechanical properties compared to glutaraldehyde, probably because genipin is covalently bound within the cross-linked matrix as previously reported [33]. The groups HGP2, HGDP3, and HGDP4 reach tensile strength values near 8 MPa, which makes them ideal candidates for several applications; however, there are no statistical differences between these groups, which demonstrates that the final hydrogel properties are not affected by these concentrations.

In Figure 4, the powder of HAp was characterized as irregular prisms 1–2 μm in diameter. In Figure 5, multiple pores can be appreciated in the hydrogel porosity, a desirable property in tissue engineering as it can promote revascularization and adequate nutrient exchange. The synthesis process did not affect the HAp, as can be noticed in Figure 5B, where the powder is seen well distributed on the surface of the hydrogel, which is an important finding because the ceramic acts as a natural binding site to promote cell adhesion.

The FTIR analysis demonstrated the cross-linking with glutaraldehyde by shift formation in the gelatin and glutaraldehyde hydrogels. Figure 6 shows a shift in the Amide II band in 1540 cm^−1^. The formation of a covalent bond between aldehydic groups of glutaraldehyde and functional groups like hydroxyl, amines, thiols, and phenols was previously reported in several works [21,35,36]. Also, the persistence of aldehyde functional groups was noticed in the hydrogel containing glutaraldehyde; those groups are highly reactive, causing cell toxicity properties [20,37]. In Figure 6B, a vibration at 1250 cm^−1^ corresponding to a Schiff base, a covalent bond between C-N, was found, demonstrating the cross-linking with the genipin in the hydrogel [30].

The gain of water is an inherited property of hydrogels; it depends on several factors as the composition, mainly the polymers and the crosslinker that form the network where water is absorbed. In Figure 7, equilibrium swelling ratio can be appreciated; the major amount of water gain occurs in the early phase, between 20 and 40 min, which could indicate a loose polymer network [38]. In the swelling percentage tests in Figure 8, it was demonstrated that the Pluronic^®^ triblock copolymer (PEG-PPG-PFG) in HGP1 and HGP2 samples can reduce the amount of water gain in the hydrogels as previously reported. Truong et al. found that triblock copolymers decrease the swelling ratio [13]. The hydrophobic properties of the PPG in the center of the copolymer can explain that finding. Additionally, hydrogels can reduce swelling in samples by increasing the number of polymers, creating a denser cross-linking network, and decreasing the mesh size of the hydrogel network [13,33]. Genipin has been shown to generate a more efficient crosslinking and a denser polymer matrix compared to glutaraldehyde; therefore, genipin hydrogels have shown less swelling capacity [39]. However, as shown in Figure 8, the Gel 2 hydrogel (gelatin and genipin) exhibited the highest swelling percentage, with statistically significant differences compared to Gel 1 (gelatin and glutaraldehyde). This could be explained by a low genipin concentration, which produces a less dense crosslinking network that gains large amounts of water as previously reported [40,41].

Only four groups were selected for the cell viability analysis. As shown in Figure 9, after 1, 3, and 5 days of cell exposure, there were no statistical differences in the absorbance against the control group TCP. However, HGD1 showed less cell viability on day 1, which can be explained by the glutaraldehyde residuals already reported as having cytotoxic capacity [20]. On day 7, significant cell proliferation was noticed, and HGP2 and HGD2 had statistical differences in contrast to TCP. Hydrogels have been described as biomaterials with suitable biological properties; they are biocompatible, and their water-gain capacity can allow oxygen and nutrient exchange [7,11]. However, excessive swelling affects their mechanical properties and cell adhesion on the hydrogels, and this could be controlled by tailoring the cross-linking [21]. In this work, the groups HGP2 and HGDP3 showed the best absorbance on day 7, which corresponds to excellent biocompatibility; the PPG polymer, which has thermo-responsive micellization that can suppress gel swelling at physiological temperature, can explain this in the formulation, leading to control of the excessive swelling capacity and therefore facilitating cell adhesion and proliferation. Also, the aim of adding HAp is to promote cell adhesion on the surface or even the inner porosity of the biomaterial and improve the swelling capacity [17].

The expression and osteogenic differentiation of MSC culture onto hydrogels were analyzed by qRTPCR test, and the expression of bone mineralization markers was quantified, as shown in Figure 10. The results showed that 1-day Col1 and ALP markers had a statistically significant expression. This can be explained because those genes can be considered part of the initial or early stages of the mineralization process, where cell proliferation is essential [42]. The osteoinduction capacity of HAp has been reported; however, the synthesis process can affect the disposition of the doped ceramic onto the hydrogels [43,44]. As can be anticipated, Figure 10B,C exhibit an increment in the SPP1 marker at day 30 against the cells with differentiation medium (TCP dif); this gene is associated with the synthesis of extracellular matrix and differentiation of MSC, confirming that the hydrogels with HAp induce the differentiation process even without cell differentiation medium. Interestingly, stiffness of the hydrogel can also induce cell differentiation by activation of mechanosensors related to transcription factor yes-associated protein (YAP)/transcriptional coactivator with PDZ-binding motif (TAZ) related to the Hippo pathway [45].

## 3. Conclusions

The present study demonstrates that genipin can synthesize biomedical scaffolds when cross-linked in the proposed composite hydrogel, improving the biological properties, as can be demonstrated against glutaraldehyde hydrogels with statistical differences at 7 days. Moreover, the results showed that hydroxyapatite was a dopant filling inorganic ceramic material that modifies the mechanical properties of hydrogels, making them stiffer and more resistant, reaching values near 8 MPa in tensile strength. Furthermore, the composite hydrogels HGDP3 and HGP2 showed the best results, demonstrated good biocompatibility and bioactivity properties, as can be appreciated by the cell viability assay and induction of MSC differentiation, which can regulate the expression of osteogenic markers without the need for differentiation medium. However, challenges still need to be addressed regarding the hydrogel proposed, and further investigation of the in vivo response needs to be done for use as a biomaterial for bone tissue engineering applications.

## 4. Materials and Methods

### 4.1. Preparation of the Different Gelatin/HAp Hydrogels

The hydrogel was synthesized following the formula employed by Obando et al. [28]. The different proportions used are reported in Table 1. PEG-D was weighed and placed in a magnetic stirrer with Phosphate buffered saline (PBS) 101866950, Sigma Aldrich, St. Louis, MO, USA) until dissolved. The mixture was then heated to 50 °C for 10 min, followed by adding type A gelatin, MP Biomedicals, LLC, 1 kg, and mixing using a Maxi Mix II vortexer. Finally, the cross-linker was added, and the mixture was heated to 50 °C for one hour. Some modifications were made to some of the hydrogels. Poly (ethylene glycol) diamine (Sigma Aldrich, St. Louis, MO, USA). (PEG-D) was replaced with Poly (ethylene glycol)-block-poly (propylene glycol)-block-poly (ethylene glycol) (PEG-PPG-PEG) Pluronic^®^ P-123 (Sigma Aldrich St. Louis, MO, USA, 435465-1L) and glutaraldehyde 2% (GA) Gludex, 1000ML was substituted with genipin (G4796-25MG Sigma Aldrich, St. Louis, MO, USA), a natural cross-linker. Additionally, the hydrogel was functionalized with Hydroxyapatite (HAp) (677418-10G nanopowder, Sigma Aldrich, St. Louis, MO, USA). Calibration curves with different concentrations of genipin (0.1, 0.5, and 1 mM) and HAp (0.005, 0.01, 0.02, and 0.04 g) were tested in the mechanical test to select the ideal hydrogels for the biological assays. Some samples of the hydrogels with the same composition and methodology were also lyophilized for SEM studies, swelling range equilibrium, and mechanical properties of the material. For this purpose, the samples were frozen at –40 °C and lyophilized in LABCONCO^®^ equipment at –47 °C with a pressure of 0.057 mBar for 24 h.

### 4.2. Mechanical Tests

Tensile strength was measured for mechanical evaluation. Ten samples of each group were prepared according to ASTM F2150-13 Standard Guide for Characterization and Testing of Biomaterial Scaffolds Used in Tissue-Engineered Medical Products [46]. Briefly, the samples of the hydrogels for the tensile test were prepared as previously described and subjected to a constant crosshead speed of 1 mm/min using a Universal Testing Machine INSTRON^®^ 5567 Norwood, MA, USA. The elastic modulus (MPa) was calculated from the linear region of the stress-strain plot, and tensile strength (MPa) was recorded as the final position of the plot.

### 4.3. Hydrogel Structure and Morphology

Due to their high-water content, hydrogel samples were lyophilized for scanning electron microscopy (SEM) observation. For this purpose, small portions of hydrogel were lyophilized as previously described. The lyophilized samples were mounted on 1 cm × 1 cm aluminum stubs using conductive carbon tape. A gold coating was applied to the samples by sputtering to ensure electrical conductivity. SEM microscopy was used to examine the ultrastructural surface morphology of the samples (JOEL JSM 7600F) at an acceleration voltage of 5 kV, using secondary electrons. ImageJ 1.53K software was used to quantify the hydrogel samples’ average pore and particle size and porosity percentage. For this purpose, the micrographs obtained by SEM were segmented, and the Image J porosity analysis tools were applied.

### 4.4. Characterization

#### 4.4.1. FTIR Analysis

The hydrogels were evaluated by Fourier Transform Infrared Spectroscopy (FTIR). An IRAffinity-1 FTIR from SHIMADZU (Kyoto, Japan) was used; 40 scans for each spectrum were collected, acquiring 1 scan per second at 5 cm^−1^ in the wavenumber range of 400–4000 cm^−1^. The absorption bands of the principal functional groups were recorded and presented in graphs.

#### 4.4.2. Equilibrium Swelling Ratio

First, the equilibrium swelling ratio was calculated because it is important to determine when hydrogels stop gaining water. Briefly, after synthesizing and lyophilizing the hydrogels, samples of ±5 mg were cut and weighed, then introduced into double-distilled water. The equilibrium in the swelling range was obtained by weighing the sample every 10 min with the following formula:

Equilibrium Swelling Ratio = (*Ws* − *Wd*)/*Wd* × 100

*Ws* is the swelling hydrogel (hydrogel weight/10 min in deionized water).
*Wd* is the dry weight (initial weight).

The test was repeated in triplicate, and the values obtained were plotted as Equilibrium Swelling Ratio versus time (min) until the values stabilized, or a plateau was reached.

#### 4.4.3. Swelling Percentage

Samples of ±5 mg of the lyophilized hydrogels were cut, weighed, and immersed in deionized water until the equilibrium swelling ratio was recorded. The swelling percentage was obtained with the formula:

Swelling Percentage = (*We* − *Wd*)/*Wd* × 100

*We* is the weight after 72 h of immersion in double-distilled water.
*Wd* is the constant dry weight.

The values obtained were used to plot the swelling percentage by comparing the groups. The test was repeated in triplicate.

### 4.5. Biological Characterization

#### 4.5.1. Cell Viability Tests

To assess the biocompatibility of the hydrogels, a cell toxicity assay was performed using Human Fetal Osteoblast 1.19 (hFOB) CRL-11372™ ATCC. The cells were cultured in an incubator at 37 °C under an atmosphere of 5% CO_2_ and 100% humidity, using α-MEM medium (Sigma-Aldrich, St. Louis, MO, USA) supplemented with 10% fetal bovine serum (FBS, Biosciences, CA, USA), 2.5 mM L-glutamine, and an antibiotic solution (streptomycin 100 μg/mL and penicillin 100 U/mL, Sigma-Aldrich, MO, USA). Cell viability was evaluated using the MTT^®^ reagent. hFOB were seeded at a density of 10 × 10^3^ cells per well in quintuplets, and cells seeded onto the Tissue Culture Plate (TCP) served as a control group. All the groups were cultured for 1, 3, 5, and 7 days. After each experimental period, the cells were incubated with 50 μL of MTT working reagent^®^ and 50 μL of phenol-free media at 37 °C for 4 h. Following the incubation period, the medium was retrieved, and 150 μL of MTT solvent reagent was added and stirred for 15 min according to the manufacturer’s instructions. The optical density was recorded at 590 nm using a Multiskan FC 96-well plate reader (Thermoscientific, Waltham, MA, USA). The absorbance values obtained were normalized to the control group (cells cultured on TCP) to represent relative cell viability.

#### 4.5.2. QRT-PCR Test

To analyze the osteogenic differentiation capacity induced by HAp hydrogels, dental pulp mesenchymal stem cells (DP-MSCs) were used as osteogenic induction models. Briefly, cells were obtained by an explant tissue culture system from adult patients who underwent third molar extraction at the Maxillofacial Surgery Clinic of the Division of Postgraduate Studies and Research (DEPel—its acronym in Spanish) at the School of Dentistry of the National Autonomous University of Mexico. Patients were informed about the study prior to obtaining their consent and donation of extracted dental organs, and the protocol was approved by the Research and Ethics Committee of the School of Dentistry (CIE/1110/2017) as previously reported [47]. The cells were cultured in a T25 flask until confluency, and 10 × 10^3^ DP-MSCs cells per well were cultured in the hydrogels for 1, 7, and 30 days to evaluate the expression of bone-associated genes at each time point using qRT-PCR. Upon reaching each time point, total RNA was extracted from cells cultured in the hydrogels using TRIzol™ Reagent (Invitrogen, Carlsbad, CA, USA). Purified RNA was synthesized into cDNA using the Improm-II™ Reverse Transcriptase system (Promega, Madison, WI, USA). The selected osteoblast marker gene expression (Table 2) was evaluated by relative qRT-PCR using the Forget-Me-Not™ EvaGreen^®^ qPCR Master Mix (Bio-Rad, Hercules, CA, USA). The GAPDH gene was employed as an internal reference control. qRT-PCR was conducted using the MyGo Pro Real-Time PCR System (Techne, Staffordshire, UK). The ΔΔCt method was utilized to calculate the relative expression of the genes of interest compared to the internal control.

### 4.6. Statistical Analysis

A normality test was carried out, and statistical analysis was performed using one-way ANOVA followed by Tukey’s post hoc test to determine significant differences between groups. All the statistical analyses were performed using GraphPad version 9.0.2 statistical software. The results were expressed as the mean ± standard deviation (SD). An asterisk (*) indicates significant differences at *p*-value < 0.05 between conditions.

## Figures and Tables

**Table 1:** Components and proportions used for the preparation of the hydrogels. PBS: Phosphate-buffered saline; PEG-D: Poly (ethylene glycol) diamine; PEG-PPG-PEG: Poly(ethylene glycol)-block-poly(propylene glycol)-block-poly(ethylene glycol); and HAp: hydroxyapatite.

	Components and Proportions						
Hydrogels	PBS	Gelatin	PEG-D	PEG-PPG-PEG	Glutaraldehyde	Genipin	HAp
Gel 1	7.8 mL	1.6 g	-	-	1.2 mL	-	-
Gel 2	7.8 mL	1.6 g	-	-	-	5 mL	-
HGD1	7.8 mL	1.6 g	0.07 g	-	1.2 mL	-	0.04 g
HGP1	7.8 mL	1.6 g	-	0.07 g	1.2 mL	-	0.04 g
HGD2	7.8 mL	1.6 g	0.07 g	-	-	5 mL	0.04 g
HGP2	7.8 mL	1.6 g	-	0.07 g	-	5 mL	0.04 g
HGDP1	7.8 mL	1.6 g	0.035 g	0.07 g	1.2 mL	-	0.04 g
HGDP2	7.8 mL	1.6 g	0.07 g	0.035 g	1.2 mL	-	0.04 g
HGDP3	7.8 mL	1.6 g	0.035 g	0.07 g	-	5 mL	0.04 g
HGDP4	7.8 mL	1.6 g	0.07 g	0.035 g	-	5 mL	0.04 g

**Table 2:** Primer sequences forward (Fw) and reverse (Rv) used in qRT-PCR.

Name	5’→3’
Glyceraldehyde-3-Phosphate Dehydrogenase (GAPDH) Fw	gcatcctgggctacactgag
Glyceraldehyde-3-Phosphate Dehydrogenase (GAPDH) Rv	tgctgtagccaaattcgttg
Collagen 1 (Col 1) Fw	gagagcatgaccgatggatt
Collagen 1 (Col 1) Rv	atgtaggccacgctgttctt
Phosphatase alkaline (ALP) Fw	cgaccagacgtgaatgagag
Phosphatase alkaline (ALP) Rv	gctacgaagctctgctcctg
Osteopontin (SPP1) Fw	cgaggtgatagtgtggtttatgg
Osteopontin (SPP1) Rv	gcaccattcaactcctcgctttc
Runt-domain transcription factor (RUNX 3) Fw	tcagcaccacaagccactt
Runt-domain transcription factor (RUNX 3) Rv	aatgggttcagttccgaggt

## Data Availability

The data presented in this study are available on request from the corresponding author.
